# NGF Upregulates the Plasminogen Activation Inhibitor-1 in Neurons via the Calcineurin/NFAT Pathway and the Down Syndrome-Related Proteins DYRK1A and RCAN1 Attenuate This Effect

**DOI:** 10.1371/journal.pone.0067470

**Published:** 2013-06-25

**Authors:** Georgios C. Stefos, Ulf Soppa, Mara Dierssen, Walter Becker

**Affiliations:** 1 Institute of Pharmacology and Toxicology, Medical Faculty of the RWTH Aachen University, Aachen, Germany; 2 Systems Biology Programme, Center for Genomic Regulation; Pompeu Fabra University, and Centro de Investigación Biomédica en Red de Enfermedades Raras, Barcelona, Spain; New York State Institute for Basic Research, United States of America

## Abstract

**Background:**

Plasminogen activator inhibitor 1 (PAI-1) is a key regulator of the plasminogen activation system. Although several lines of evidence support a significant role of PAI-1 in the brain, the regulation of its expression in neurons is poorly understood. In the present study we tested the hypothesis that NGF induces the upregulation of PAI-1 via the calcineurin/nuclear factor of activated T cells (NFAT) pathway and analysed whether the overexpression of the Down syndrome-related proteins DYRK1A and RCAN1 modulated the effect of NGF on PAI-1 expression.

**Results:**

NGF upregulated PAI-1 mRNA levels in primary mouse hippocampal neurons cultured for 3 days *in vitro* and in the rat pheochromocytoma cell line PC12. Reporter gene assays revealed that NGF activated the calcineurin/NFAT pathway in PC12 cells. Induction of PAI-1 by NGF was sensitive to the calcineurin inhibitor FK506 and the specific inhibition of NFAT activation by the cell permeable VIVIT peptide. Activation of calcineurin/NFAT signalling through other stimuli resulted in a much weaker induction of PAI-1 expression, suggesting that other NGF-induced pathways are involved in PAI-1 upregulation. Overexpression of either DYRK1A or RCAN1 negatively regulated NFAT-dependent transcriptional activity and reduced the upregulation of PAI-1 levels by NGF.

**Conclusion:**

The present results show that the calcineurin/NFAT pathway mediates the upregulation of PAI-1 by NGF. The negative effect of DYRK1A and RCAN1 overexpression on NGF signal transduction in neural cells may contribute to the altered neurodevelopment and brain function in Down syndrome.

## Introduction

The serine protease inhibitor plasminogen activator inhibitor-1 (PAI-1) is a component of the plasminogen activation system. PAI-1 forms stable complexes with both tissue plasminogen activator (tPA) and urokinase plasminogen activator (uPA) and thus prevents the activation of plasminogen to plasmin [1].

PAI-1 is expressed in a variety of tissues including the brain [2]. As a regulator of plasminogen activation, PAI-1 is involved in the degradation of the extracellular matrix, which is important for synaptic remodelling, as well as in the regulation of the plasmin-mediated cleavage of proBDNF to BDNF [3]. As a tPA inhibitor, PAI-1 protects neurons from the tPA potentiation of the NMDA-induced calcium influx and necrosis [4]. PAI-1 has also a neurotrophic/neuroprotective role in PC12 cells, which has been suggested to be independent of the plasminogen activation system [5]. During the last decade PAI-1 has also attracted increasing attention for its potential function in Alzheimer disease both as a neurotrophic factor and as an inhibitor of plasmin’s α-secretase cleavage activity [5], [6].

Dual-specificity tyrosine phosphorylation-regulated kinase 1A (DYRK1A) is a protein kinase member of the DYRK family that phosphorylates serine and threonine residues in its substrates and autophosphorylates a tyrosine residue in its activation loop [7]. The human *DYRK1A* gene is located on chromosome 21 [8] and its overexpression is considered to be related to Down syndrome phenotypes. Both murine *Dyrk1A* and human *DYRK1A* genes are expressed in several regions of the brain [9], [10], [11], where they play a role in neuronal development [12] and adult brain function [13]. Transgenic mice that carry an extra copy of the *Dyrk1A* gene exhibit neurodevelopmental delay, motor abnormalities and cognitive deficits that resemble those described in Down syndrome individuals [14], [15], [16].

Regulator of calcineurin 1 (RCAN1), which was formerly known as DSCR1 or calcipressin 1, binds to and inhibits the protein phosphatase calcineurin and its signalling pathways [17]. The *RCAN1* gene is also located on the human chromosome 21, is highly expressed in the human brain [18] and is considered to be associated with Down syndrome traits. RCAN1 overexpression in transgenic mice affects the visuo-spatial learning and memory tasks and causes structural brain abnormalities in those areas affected in DS [19], [20].

NFAT is a family of transcription factors consisting of the five members NFAT1–5. Except for NFAT5 all of them are calcium-regulated [21]. In the basal state the regulatory domain of NFAT is heavily phosphorylated and thus the transcription factor is inactive and localized in the cytoplasm. Rise in intracellular Ca2+ results in the activation of the calmodulin-dependent phosphatase calcineurin, which in turn dephosphorylates NFAT. After dephosphorylation, a nuclear localization sequence is exposed and NFAT translocates to the nucleus where it binds to target DNA sequences either as homodimers, heterodimers or through interaction with other transcription factors [22], [23], [24]. Benedito et al. [25] showed that NFAT3 plays a critical role in mediating survival of granule neurons of the developing cerebellum and Graef et al. [26] showed that NFATc2/c3/c4 null mice have defects in axon outgrowth.

DYRK1A and RCAN1 are negative regulators of NFAT activity. RCAN1 affects NFAT localization by inhibiting the enzymatic activity of calcineurin [27] and DYRK1A phosphorylates NFAT and promotes its nuclear export by priming the subsequent phosphorylation by GSK3 [28], [29]. Furthermore, DYRK1A was shown to interact with RCAN1, extending its half life and enhancing its binding on calcineurin [30]. Taken together, DYRK1A and RCAN1 can act synergistically to block NFAT-dependent transcription, suggesting that the reduced NFAT activity resulting from the 1.5-fold overexpression of DYRK1A and RCAN1 could be the basis of many features of Down syndrome [28], [31].

In the PC12 cell line, PAI-1 is induced by NGF [32], [33]. Furthermore, NGF causes Ca2+ mobilization and consequently NFAT activation in several cell lines [34], [35]. Given that Down syndrome individuals have reduced PAI-1 in blood compared with controls [36], the aim of the present study is: a) to test the hypothesis that the calcineurin/NFAT pathway is involved in the NGF-induced upregulation of PAI-1 in neuronal cells and b) to analyse whether the overexpression of the Down syndrome-related proteins DYRK1A and RCAN1 can regulate the NGF effect on PAI-1 by modulating NFAT deactivation.

## Results

### NGF Induces PAI-1 Expression

**Figure 1:**
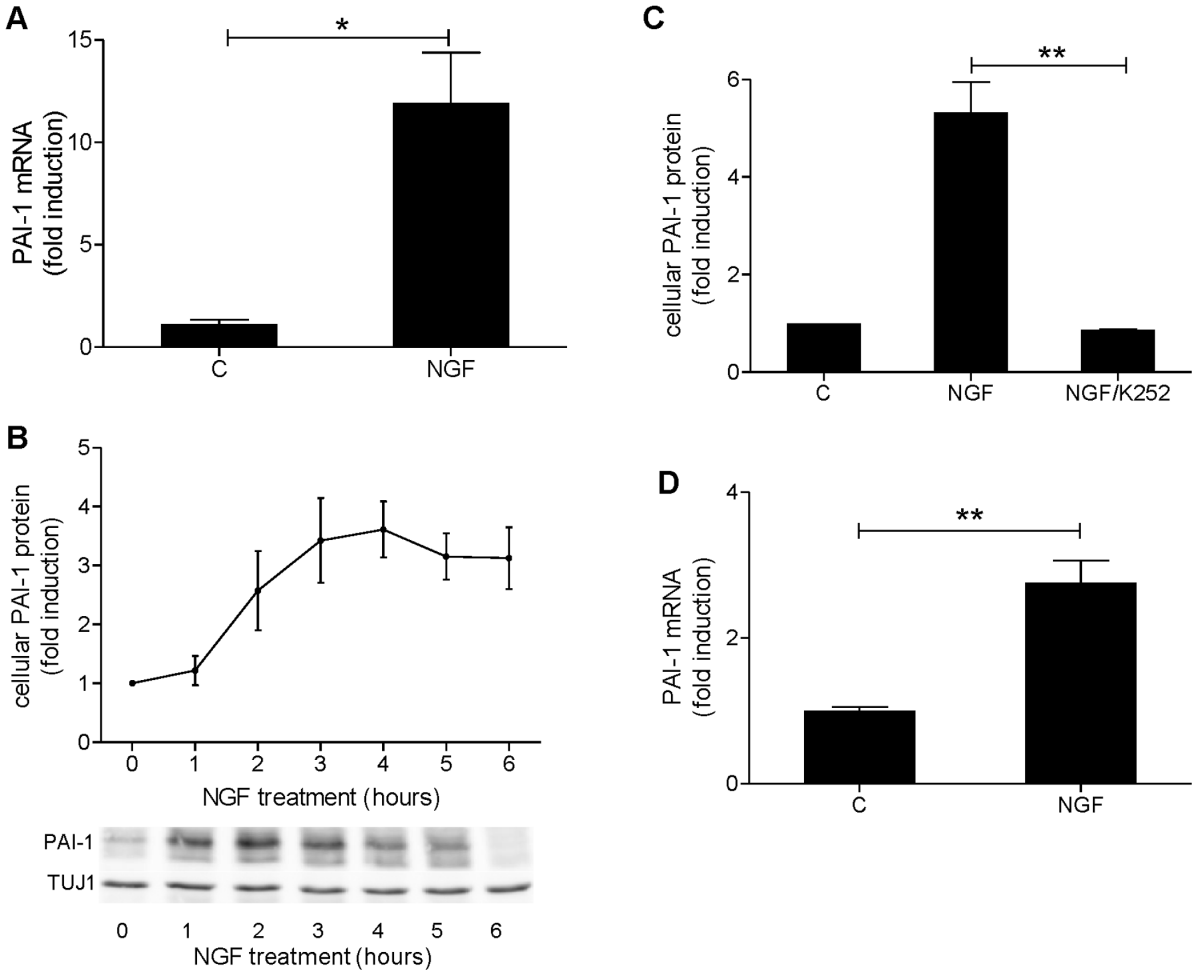
NGF induces the expression of PAI-1. A, PC12 cells were treated for 2.5 h with 50 ng/ml NGF or were not treated (C). PAI-1 mRNA was quantified by qRT-PCR. B, PC12 cells were treated with 50 ng/ml NGF for the times indicated. PAI-1 levels in total cell lysates were determined by quantitative evaluation of immunoblots as shown in the lower panel. The upper one of the double band represents the active form of PAI-1, while the lower band represents the cleaved form. Intensities of both bands were combined for quantification and normalized to the TUJ1 signal. C, PC12 cells were treated with 50 ng/ml NGF for 3 h in the presence or absence of 300 nM K252A. The inhibitor was added 30 min before NGF. PAI-1 protein levels in total cell lysates were determined by quantitative evaluation of immunoblots. D, Primary hippocampal cultures from mouse embryos at DIV3 were treated for 2 h with 100 ng/ml NGF before PAI-1 mRNA levels were quantified by qRT-PCR. The diagrams present means ± SEM from 3 independent experiments. C, Control; *, p<0.05; **, p<0.01.

PC12-NFAT-Luc cells expressing luciferase under the control of an NFAT-driven promoter [37] were used for the current study and will be referred to as PC12 throughout. NGF was tested for its ability to induce PAI-1 in this PC12 subclone and resulted in a 12-fold induction of PAI-1 mRNA levels (Fig. 1A). In a time-course experiment (Fig. 1B) the cellular (non-secreted) PAI-1 protein levels increased after 2 h of NGF treatment and reached the maximum level after 3–4 h. The Trk inhibitor K252a abolished the effect of NGF on PAI-1 protein levels (Fig 1C), suggesting that NGF exerts its effect via the TrkA receptor. Next we asked whether NGF induces PAI-1 expression also in primary neurons. Cortical and hippocampal neuronal cultures from mouse embryos at embryonic day 17 (E17) were prepared. On day *in vitro* 3 (DIV3) treatment with NGF for 2 h increased the PAI-1 mRNA levels about 2.5-fold in hippocampal cultures (Fig. 1D) but had no effect on cortical cultures (data not shown). At later stages of the culture (DIV14) NGF had no effect on PAI-1 expression in either hippocampal or cortical cultures (data not shown).

### NGF Upregulates PAI-1 *via* the Calcineurin/NFAT Pathway

**Figure 2:**
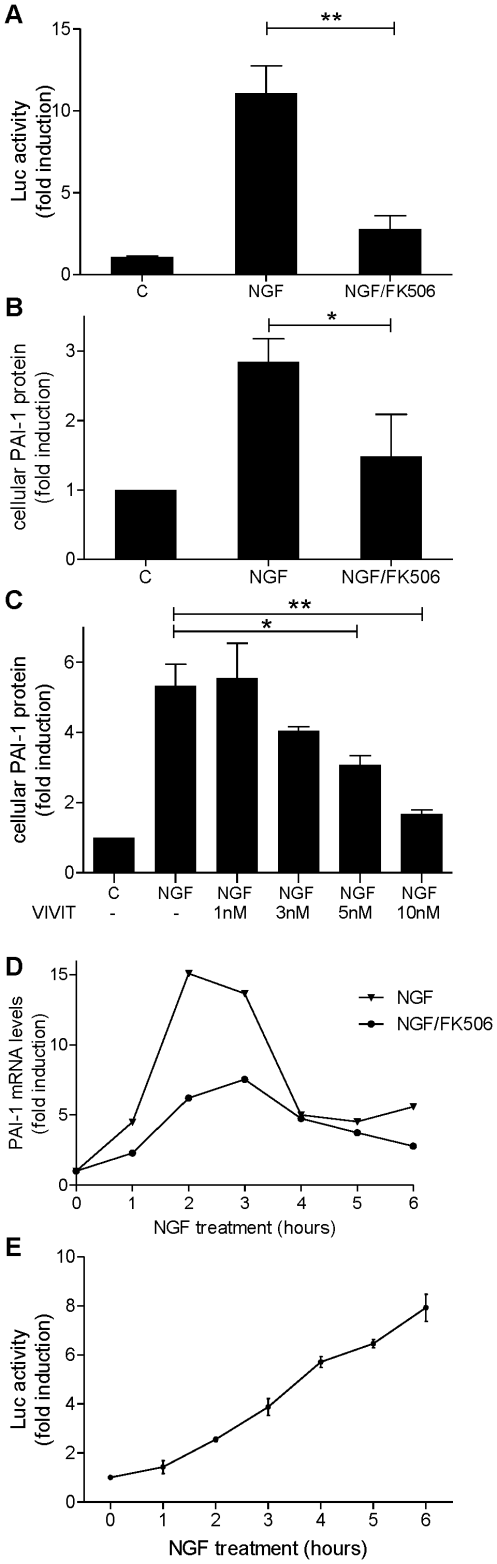
NGF upregulates PAI-1 *via* the calcineurin/NFAT pathway. PC12 cells were maintained in the presence or absence of NGF (50 ng/ml) for 3 h. When indicated, FK506 (10 nM) or VIVIT (concentrations are indicated) were added 30 min before NGF. Cell lysates were assayed for NFAT-driven luciferase activity (A,E) or cellular PAI-1 levels (B,C). PAI-1 mRNA was determined by qRT-PCR (D). The diagrams in panels A, B, C and E present means ± SEM from 3 independent experiments and the graph in panel D shows one representative experiment of 2 replicates. C, Control; *, p<0.05; **, p<0.01.

To examine whether NGF can activate NFAT in our cell model, PC12-NFAT-Luc cells were treated with NGF in the presence or absence of FK506, a specific inhibitor of calcineurin [38]. NGF increased the NFAT-dependent promoter activity about 11-fold, while FK506 reduced this effect by 75% (Fig. 2A). Next we tested whether calcineurin mediates the NGF-derived upregulation of PAI-1. As shown in figure 2B, NGF upregulated the cellular PAI-1 protein about 3-fold and FK506 reduced by 50% the PAI-1 levels in NGF-treated cells. To further prove the involvement of the NFAT, we used the cell permeable VIVIT peptide, which selectively interferes with the calcineurin/NFAT interaction and thereby inhibits NFAT activation without affecting calcineurin catalytic activity [39]. Figure 2C shows that VIVIT inhibited the NGF effect on PAI-1 protein levels. To confirm that NGF upregulates PAI-1 by transcriptional regulation, the PAI-1 mRNA levels were quantified in a time-course experiment. FK506 partially inhibited the NGF-induced increase of PAI-1 transcripts and this inhibition was maximal around the time point that PAI-1 mRNA levels peaked (Fig. 2D). Throughout the experiment NGF constantly induced NFAT promoter activity (Fig. 2E) as reflected by the linear accumulation of luciferase over time.

### NFAT Activation is not Sufficient for NGF-dependent PAI-1 Induction in PC12 Cells

**Figure 3:**
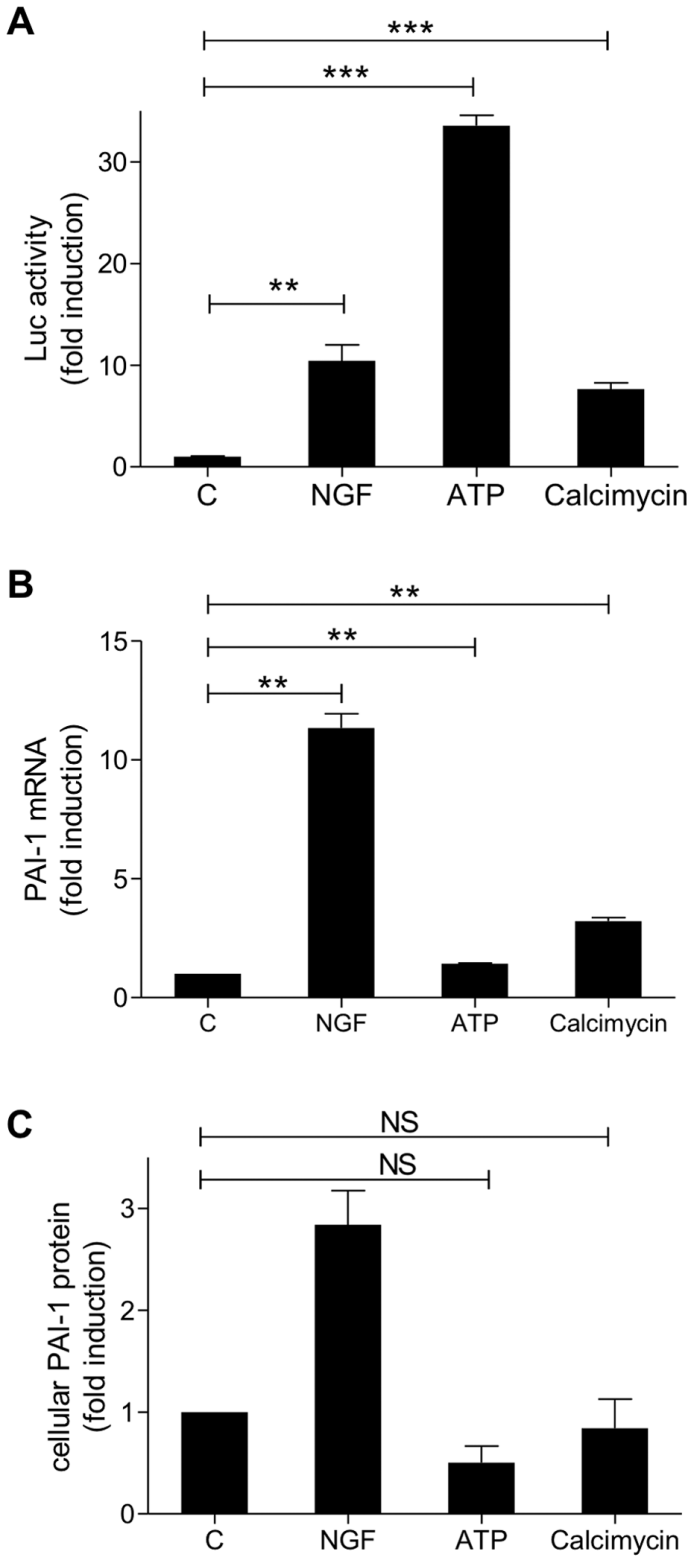
Activation of NFAT is not sufficient for maximal upregulation of PAI-1. PC12 cells were treated with NGF (50 ng/ml), ATP (300 µM) or calcimycin (10 µM) for 3 h before cell lysis. Cell lysates were assayed for NFAT dependent reporter gene (A), PAI-1 mRNA levels (B) or cellular PAI-1 protein levels (C). The diagrams show the means ± SEM from 3 independent experiments. C, Control; NS, not significant; **, p<0.01; ***, p<0.001.

To further elucidate the contribution of activated NFAT on PAI-1 expression, PC12 cells were treated with ATP, which causes Ca2+ influx *via* P2X receptors, or the ionophore calcimycin. These stimuli increase the intracellular Ca2+ level and thereby activate the NFAT-mediated gene transcription in PC12 cells [37], [40]. Both treatments activated NFAT-dependent promoter activity in our cell system and slightly increased PAI-1 transcription but not PAI-1 protein levels (Fig. 3). However, NGF produced a much greater induction of PAI-1 expression than ATP, although ATP was the strongest inducer of NFAT transcriptional activity in the promoter assay. This result indicates that pathways other than calcineurin/NFAT must be involved in the induction of PAI-1 expression by NGF. The finding of a weaker increase of PAI-1 protein levels as compared to mRNA levels may be due to the different methodology or may reflect effects of translational and post translational regulation.

### Potential Role of PKC in the NGF-driven Induction of PAI-1 in PC12 Cells

**Figure 4:**
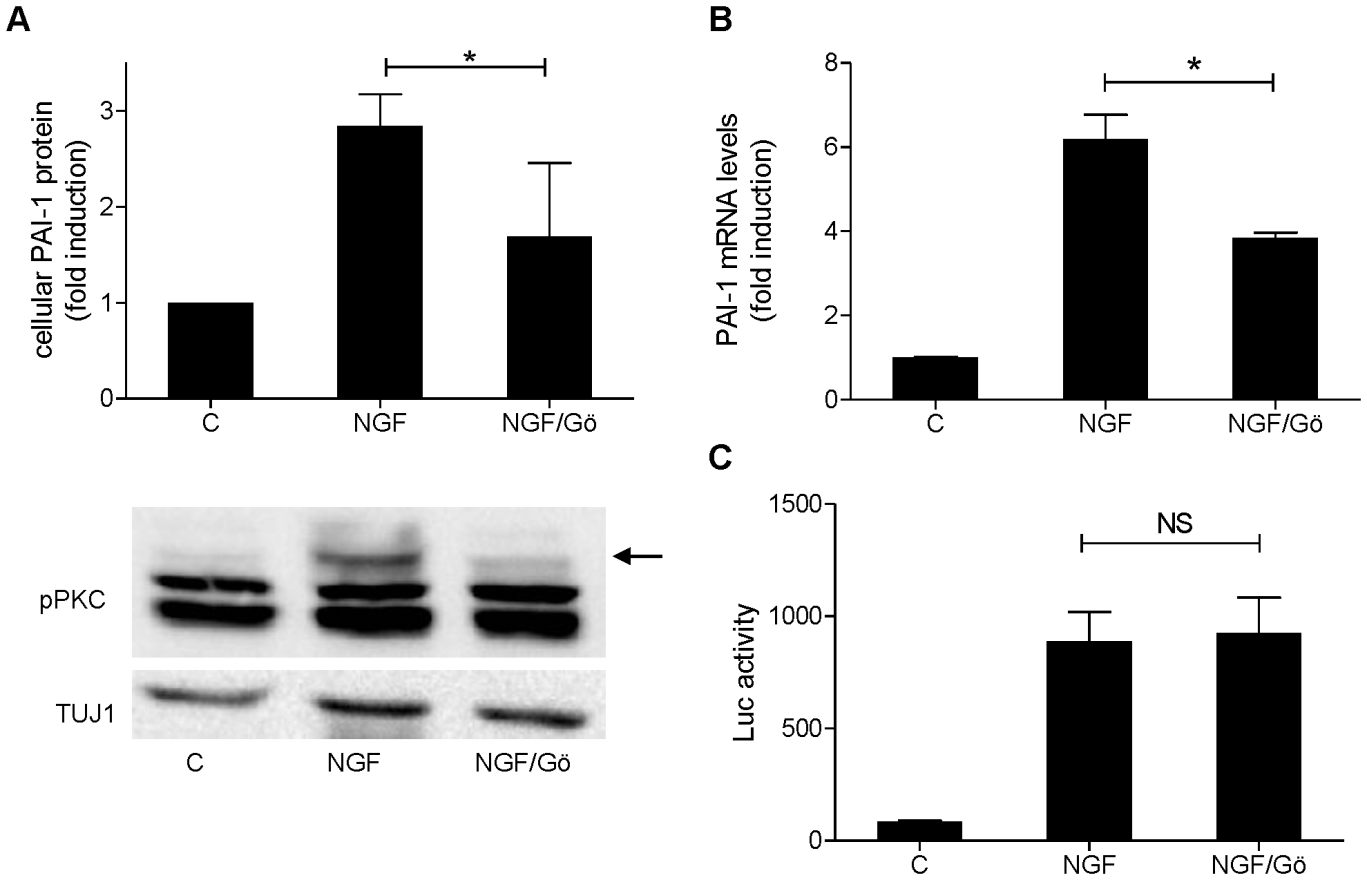
Implication of PKC in the regulation of PAI-1 by NGF. PC12 cells were treated with NGF (50 ng/ml) for 2.5 h (B) or 3 h (A, C). Gö6976 (7.5 µM) was added 30 min before NGF treatment. A, Cellular PAI-1 levels (upper panel) and autophosphorylation of PKC isoforms (lower panel). B, PAI-1 mRNA levels. C, NFAT dependent reporter gene activity. Diagrams present means ± SEM from 3 independent experiments. C, Control; NS, not significant; *, p<0.05.

Since NGF is known to activate PKC [41] and because PAI-1 expression has been shown to be regulated by PKC in other cell types [42], we asked whether PKC was involved in the NGF-induced upregulation of PAI-1 in PC12 cells. Treatment of PC12 cells with the PKC inhibitor Gö6976 reduced the effect of NGF on both PAI-1 protein and mRNA levels (Fig. 4A upper panel, 4B) without affecting NFAT activation (Fig. 4C), confirming the notion that PAI-1 induction by NGF depends on a pathway separate from NFAT activation. We used a phospho-specific PKC antibody, which detects only the autophosphorylated/activated forms of most PKC isoforms, to monitor the activation of PKC by NGF. NGF altered the phosphorylation pattern of at least one of the PKC isoforms, as shown by the strong increase of the most slowly migrating band (Fig. 4A lower panel, arrowhead). This increase was reduced by treatment with Gö6976.

### DYRK1A Overexpression Counteracts the Effect of NGF on PAI-1

**Figure 5:**
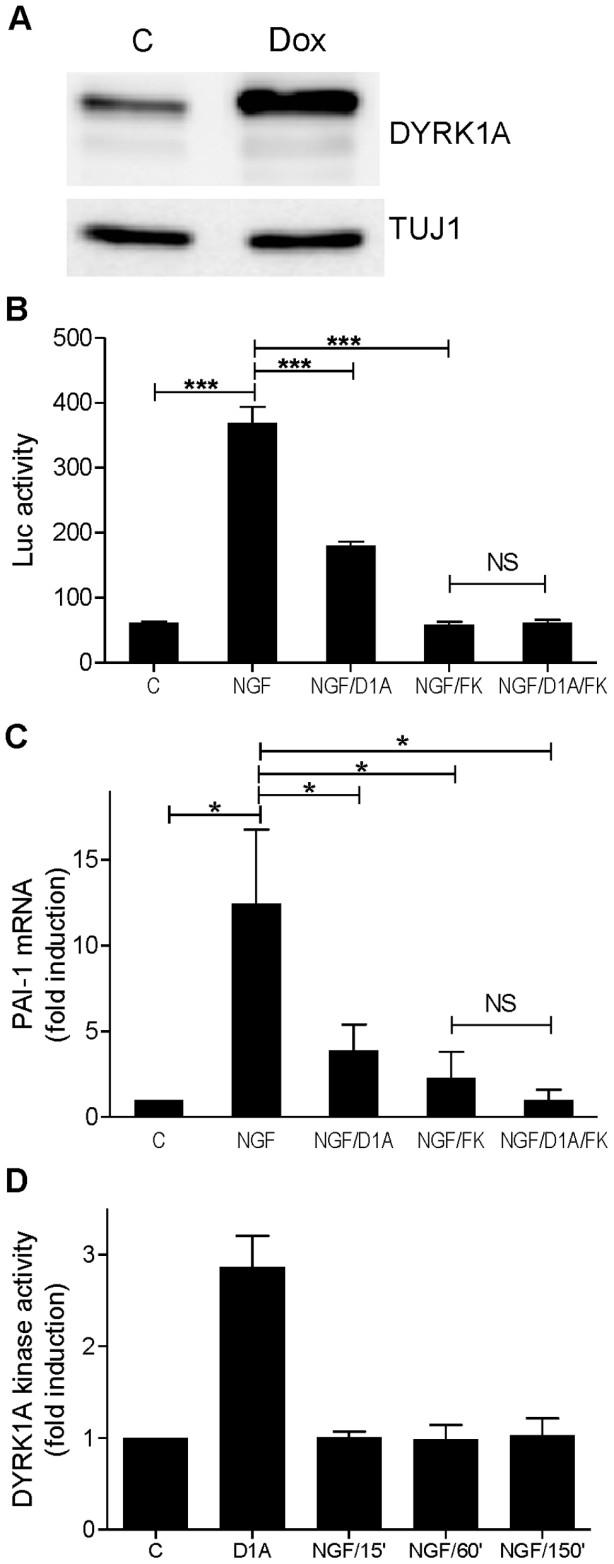
DYRK1A overexpression reduces the NGF effect on PAI-1 expression. PC12 cells that allow conditional overexpression of DYRK1A under the control of doxycyclin were generated. DYRK1A overexpression was induced by treating the cells with doxycyclin overnight. Cells were treated with NGF (50 ng/ml) for 2.5 h (C) or for 3 h (B) or for the indicated times (D). FK506 (10 nM) was added 30 min before NGF treatment. A, Western blot analysis showing DYRK1A overexpression. B, Effect of DYRK1A overexpression on NFAT-driven promoter activity. C, Effect of DYRK1A overexpression on PAI-1 transcripts levels. D, DYRK1A kinase activity was determined by immunocomplex kinase assays in untreated PC12 cells (C) or after cells were treated with NGF for 15, 60 or 150 min. Doxycyclin-induced PC12 cells overexpressing DYRK1A were used as a positive control (D1A). Column diagrams present the means ± SEM from 3 independent experiments. C, control; D1A, DYRK1A overexpression was induced by overnight treatment with doxycyclin; FK, FK506; NS, not significant; *, p<0.05; ***, p<0.001.

Given that NGF upregulated PAI-1 in an NFAT-dependent manner and that DYRK1A phosphorylates NFAT and promotes its nuclear export [29], we examined the potential effect of DYRK1A on the NGF-induced PAI-1 expression. PC12-NFAT-Luc cells were cotransduced with the FUW-tetO-DYRK1A and FUW-M2rtTA lentiviruses in order to conditionally overexpress DYRK1A under the control of a doxycycline dependent promoter. Treatment with doxycycline resulted in about 3-fold higher levels of DYRK1A in these cells (Fig. 5A).

**Figure 6:**
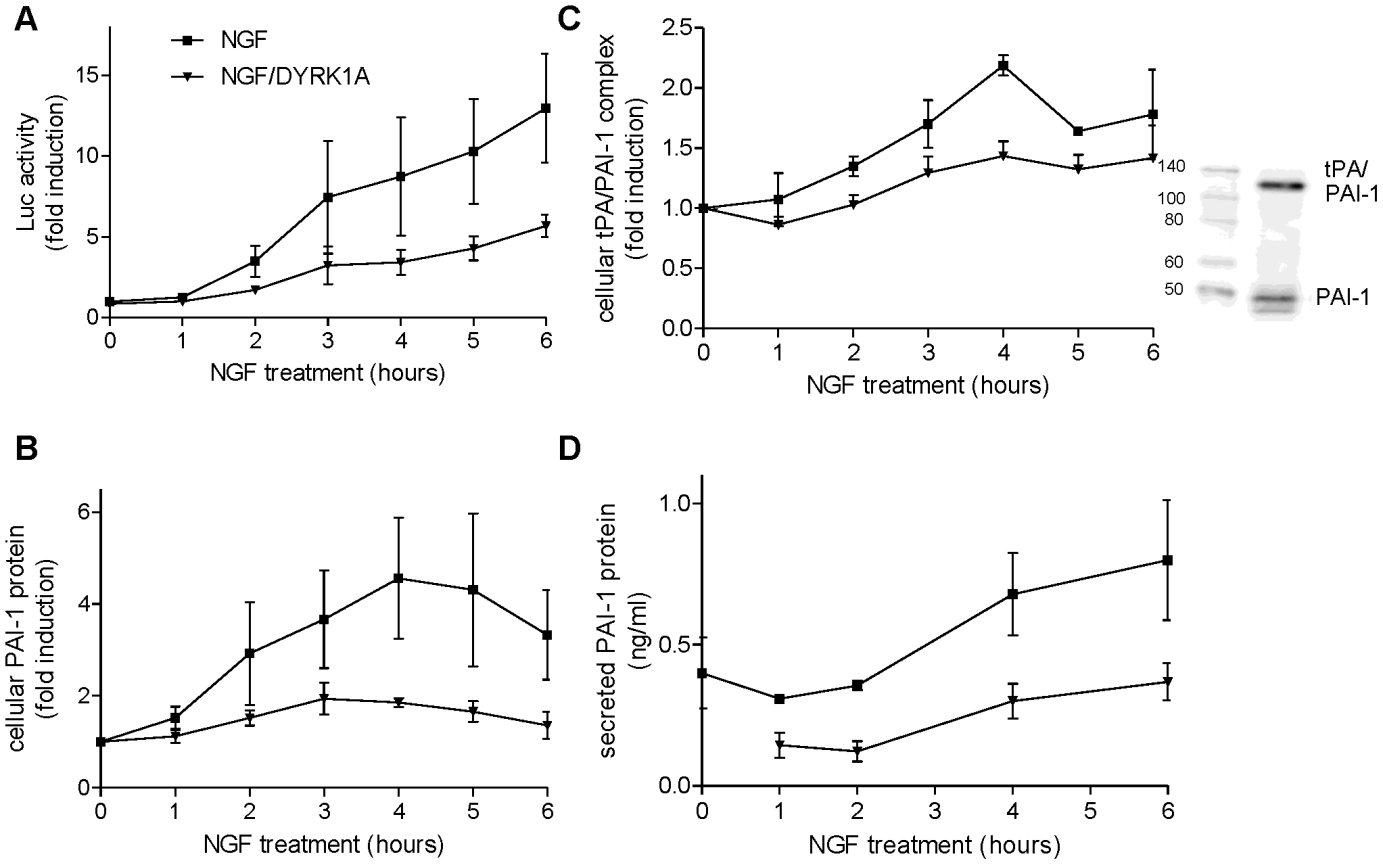
Effect of DYRK1A overexpression on the NGF-induced activation of NFAT and the upregulation of PAI-1. PC12 cells that conditionally overexpress DYRK1A were treated with NGF (50 ng/ml) for variable times. Quantifications were done on indicated times on cell lysates (A-C) or aliquots of the medium (D). Doxycycline was added overnight before NGF treatment when indicated as NGF/DYRK1A. A, Quantification of NFAT promoter activity by luciferase assays. B, Quantification of the cellular PAI-1 protein levels by immunoblotting. C, Quantification of the cellular tPA/PAI-1 complex levels by immunoblotting. The right panel shows a typical western blot for cellular PAI-1 and tPA/PAI-1 complex. D, Quantification of secreted PAI-1 protein levels in the culture medium by ELISA. Means ± SEM from 3 independent experiments are presented. The treatment effects were statistically significant as judged by comparison of the areas under the curve (p<0.05; Student’s test).

Figure 5B shows that the overexpression of DYRK1A inhibited the NGF induced activation of the NFAT by more than 60%. FK506 totally blocked the NGF effect, while DYRK1A overexpression had no additional effect. Similarly, DYRK1A overexpression reduced by 75% the NGF-induced increase of PAI-1 mRNA levels (Fig. 5C). Overexpression of DYRK1A resulted in a 3-fold increase of DYRK1A kinase activity, whereas NGF treatment had no effect on DYRK1A activity in PC12 cells (Figure 5D). Throughout the 6-hours time-course experiment, DYRK1A overexpression partially blocked the NFAT activation (Fig. 6A) and reduced the cellular PAI-1 protein levels (Fig. 6B). Next we investigated whether DYRK1A overexpression also reduced the amount of cellular tPA/PAI-1 complexes and the free PAI-1 secreted by the cell. Serpin class protease inhibitors like PAI-1 form covalent complexes with their cognate proteases that do not dissociate during SDS-PAGE [43] (Fig. 6C, right panel). Quantitative evaluation of the bands corresponding to the tPA/PAI-1 complex and of total secreted PAI-1 in the supernatant (free, latent and complexed) suggested that all forms of PAI-1 were reduced by DYRK1A overexpression (Fig. 6C and D). Cellular PAI-1 levels showed the highest NGF effects, probably because the major part of secreted PAI-1 is trapped in cellular tPA/PAI-1 complexes (see panel C).

### RCAN1 Overexpression Decreases the Effect of NGF on PAI-1 Expression

**Figure 7:**
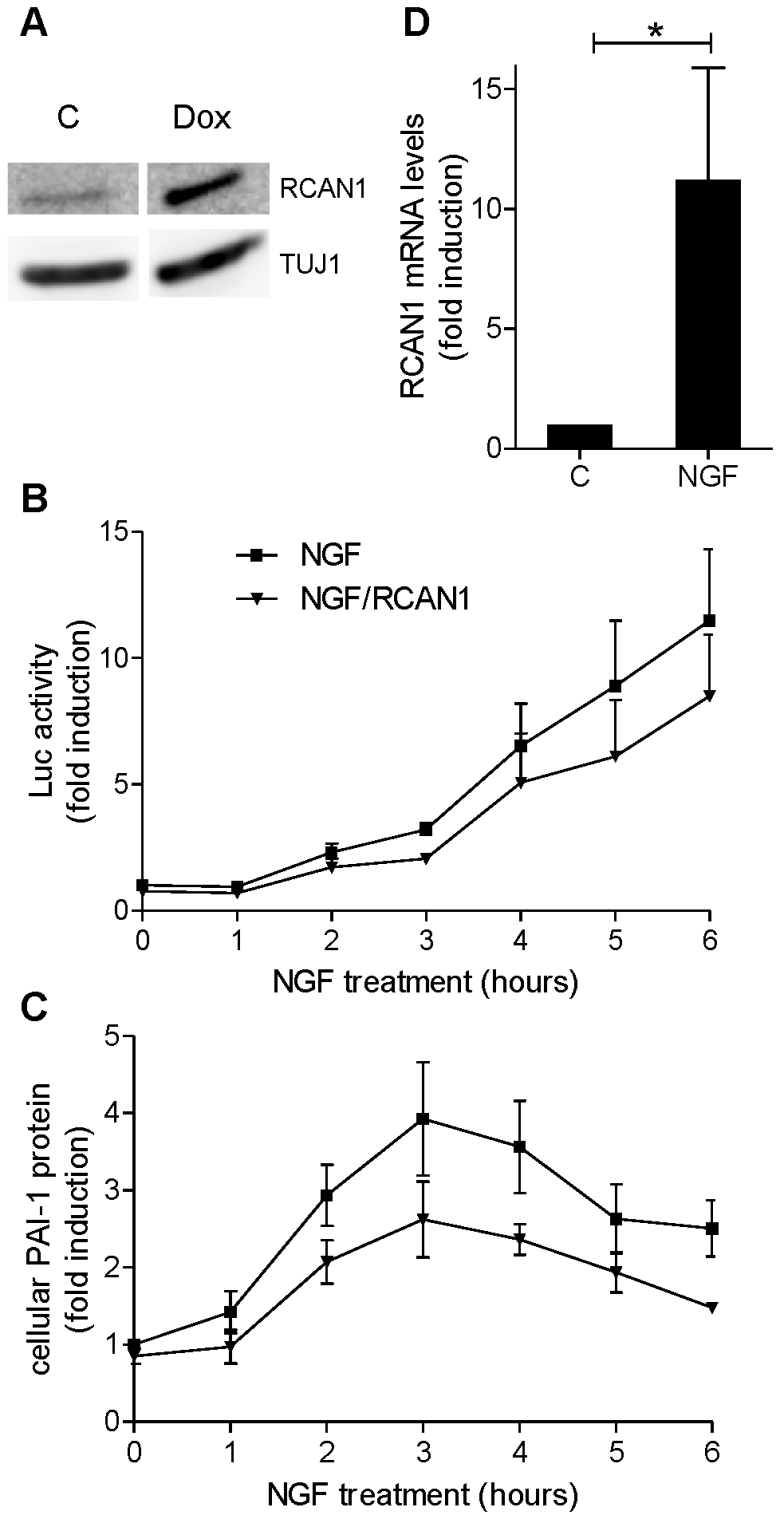
Effect of RCAN1 overexpression on the NGF-induced activation of NFAT and the upregulation of PAI-1. PC12 cells that conditionally overexpress RCAN1 (A,B and C) were treated with NGF (50 ng/ml) for the times indicated. Doxycycline was added overnight before NGF treatment in the cells indicated as NGF/RCAN1. A, Western blot analysis showing RCAN1 overexpression. B, Quantification of NFAT promoter activity by luciferase assays. C, Quantification of the cellular PAI-1 protein levels by immunoblotting. D, PC12 cells were treated with NGF (50 ng/ml) for 2.5 h and the RCAN1.4 transcript levels were quantified by qRT-PCR. *, p<0.05; C, control. Means ± SEM from 3 independent experiments are presented. The treatment effects in panels B and C were statistically significant as judged by comparison of the area under the curve (p<0.05; Student’s t test).

Since RCAN1 is also a regulator of NFAT activity, we next studied the effect of RCAN1 overexpression on the NGF-induced PAI-1 expression. PC12 cells were transduced with the FUW-tetO-HA.RCAN1 and FUW-M2rtTA lentiviruses. Figure 7A shows that doxycycline treatment resulted in about 3-fold overexpression of RCAN1. Throughout a time-course experiment RCAN1 overexpression reduced the NFAT promoter activity (Fig. 7B) and the cellular PAI-1 protein levels (Fig. 7C). Since the transcription of RCAN1 is known to be affected by NFAT [37], we next asked whether in addition to PAI-1, RCAN1 was also upregulated by NGF in PC12 cells. Figure 7D shows that NGF induces the transcription of RCAN1.

### DYRK1A Affects the Differentiation of PC12 Cells

**Figure 8:**
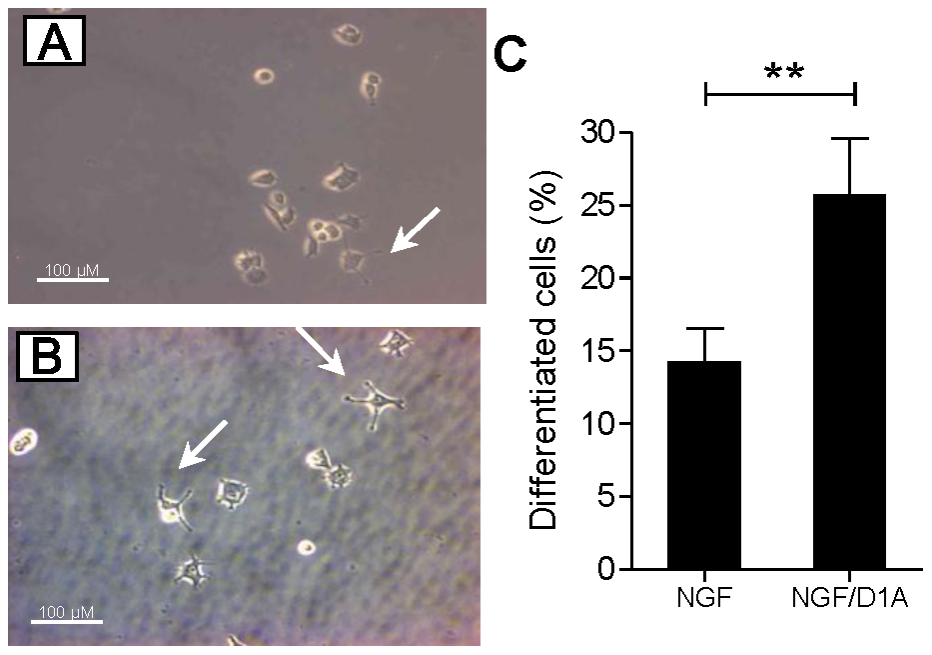
Effect of DYRK1A on PC12 differentiation. Differentiation of PC12 cells that conditionally overexpress DYRK1A was induced as described in the methods section by treatment with NGF (50 ng/ml) for 48 h in the presence or absence of doxycycline. A, B, Bright field microscopy of control (A) and DYRK1A overexpressing (B) cells. Arrows point to cells that were counted as positive. C, Quantification of differentiated cells. Means ± SEM from 3 independent experiments are presented. **, p<0.001; scale bar, 100 µM; D1A, DYRK1A overexpression.

To test whether DYRK1A overexpression influences the NGF-induced neuronal differentiation of PC12 cells, cells were treated with NGF in the presence/absence of doxycycline for DYRK1A overexpression (Fig. 8A, 8B). After 2 days the fraction of the cells with one or more neurites at least 1.5-time longer than the cell soma was determined. Figure 8C shows that DYRK1A overexpression promoted PC12 differentiation by about 40%.

## Discussion

The present study shows that NGF regulates gene expression in neuronal cells through the calcineurin/NFAT pathway and that this effect is modulated by proteins that are overexpressed in Down syndrome. Specifically, we show that NGF induces PAI-1 expression in the neural cell line PC12 *via* the activation of NFAT and that overexpression of DYRK1A or RCAN1 counteracts the NGF-induced activation of NFAT and upregulation of PAI-1 expression.

### NGF Induces PAI-1

The NGF induction of PAI-1 expression in the neural cell line PC12 cells has been previously described [32], [33]. Extending the previous observation that this effect was inhibited by the unspecific tyrosine kinase inhibitor genistein [33], we found that it was sensitive to the TrkA inhibitor K252a. This pathway was also active in cultured murine neurons. NGF upregulates PAI-1 in hippocampal neurons during early neuritogenesis (DIV3) but not during the beginning of synaptogenesis (DIV14). A putative role of PAI-1 during neuronal differentiation is supported by the observation that treatment with PAI-1 promoted the NGF-induced differentiation of PC12 cells [44]. Furthermore, given the documented role of the plasminogen activation system in cell migration, PAI-1 may also be involved in the migration of neuronal progenitors in the hippocampus [45].

### NGF Induces PAI-1 *via* the Calcineurin/NFAT Pathway

The NGF-induced upregulation of PAI-1 expression was sensitive to the calcineurin inhibitor FK506 and the VIVIT peptide, a specific inhibitor of NFAT activation. These results provide clear evidence that the calcineurin/NFAT pathway is critical for this gene regulatory effect of NGF. Furthermore, NGF robustly activated NFAT-dependent promoter activity in the PC12 cells and induced the expression of the NFAT-responsive RCAN1-4 mRNA. A previous study by Minneman *et al*. [46] had observed no effect of NGF on NFAT-driven promoter activity in PC12 cells, possibly due to differences between the PC12 subclones used in the studies.

Although NFAT is a key transcriptional regulator of neuronal development and function, it is poorly characterized as a downstream target of NGF in neurons. Groth e*t al*. [35] showed that NGF increased the NFAT-dependent transcription of BDNF and COX-2 in dorsal root ganglion cells, whereas Nguyen et al. [47] reported that NGF repressed the expression of GAP-43 (growth associated protein 43) in PC12 cells and in cultured cortical neurons through NFAT3. Our results support the hypothesis that the calcineurin/NFAT pathway mediates gene regulatory effects of NGF in neurons.

### Calcineurin/NFAT Pathway is not Sufficient for Maximal PAI-1 Induction

Treatment with calcimycin alone was previously shown to increase PAI-1 secretion in the human U937 lymphoma and HepG2 hepatoma cell lines [48], [49]. Interestingly, ATP and calcimycin-induced NFAT activation did not increase PAI-1 expression to the same degree as NGF. This result suggests that additionally to the activation of NFAT1-4 (the calcium-responsive NFAT isoforms) non calcium-responsive pathways must be activated for the maximal upregulation of PAI-1 in PC12 cells.

### A Putative Role of PKC

NGF induced the activation of at least one PKC isoform in PC12 cells (Fig. 4A). Both PKC activation and PAI-1 induction but not NFAT activation were blocked by Gö6976, which inhibits PKCα and PKCβ1 but does not inhibit PKCδ, PKCε and PKCδεζ (IC50>>3 µM [50]). These results suggest that PKC is also involved in the regulatory effect of NGF on PAI-1 expression. However, it must be noted that other kinases such as MSK1 and CHK1 are also sensitive to this inhibitor [51]. Studies in several cell types support the idea that PAI-1 regulation is often mediated by activation of multiple signalling pathways and that PKC is often one of them [52], [53], [54]. In accordance with the effect of Gö6976 in PC12 cells, PKCα [55] and PKCβ [56], [57] have been shown to regulate PAI-1 expression in other cell types.

### DYRK1A and RCAN1 Modulate the NGF-induced Upregulation of PAI-1

We show here that the overexpression of the Down syndrome-related genes *Dyrk1A* and *Rcan1* reduce the effect of NGF on NFAT promoter activity and the upregulation of PAI-1 in PC12 cells. DYRK1A negatively regulates the calcineurin/NFAT-mediated transcription by phosphorylating NFAT and thereby promoting its nuclear export [28], [29]. RCAN1 has a dual effect on calcineurin/NFAT signalling by functioning as an inhibitor or a facilitator depending on its cellular levels [58], [59], [60]. RCAN1 executes its inhibitory role by directly binding calcineurin and inhibiting the dephosphorylation of NFAT [17]. In PC12 cells NGF had no effect on DYRK1A kinase activity but increased the mRNA levels of RCAN1-4, suggesting the existence of a mechanism of negative feedback for the NFAT activation. Both *DYRK1A* and *RCAN1* are overexpressed in neurons trisomic for chromosome 21 [61], [62]. Furthermore, DYRK1A has recently been reported to phosphorylate RCAN1 and to enhance its ability to inhibit calcineurin [30]. This is in line with the suggestion that the simultaneous overexpression of both proteins in Down syndrome inhibits NFAT signalling in a cooperative manner [31].

### Effect of DYRK1A on PC12 Differentiation

Overexpression of DYRK1A reduced the NGF-induced activation of NFAT but promoted the NGF-induced differentiation of PC12 cells. This result suggests that maximal activation of the NFAT is not essential for the differentiation effect of NGF. Indeed, a previous study showed that DYRK1A overexpression potentiates NGF-mediated PC12 neuronal differentiation by up-regulating the Ras/MAP kinase signaling pathway [63].

### Implications for the Role of the Pathway in Down Syndrome

**Figure 9:**
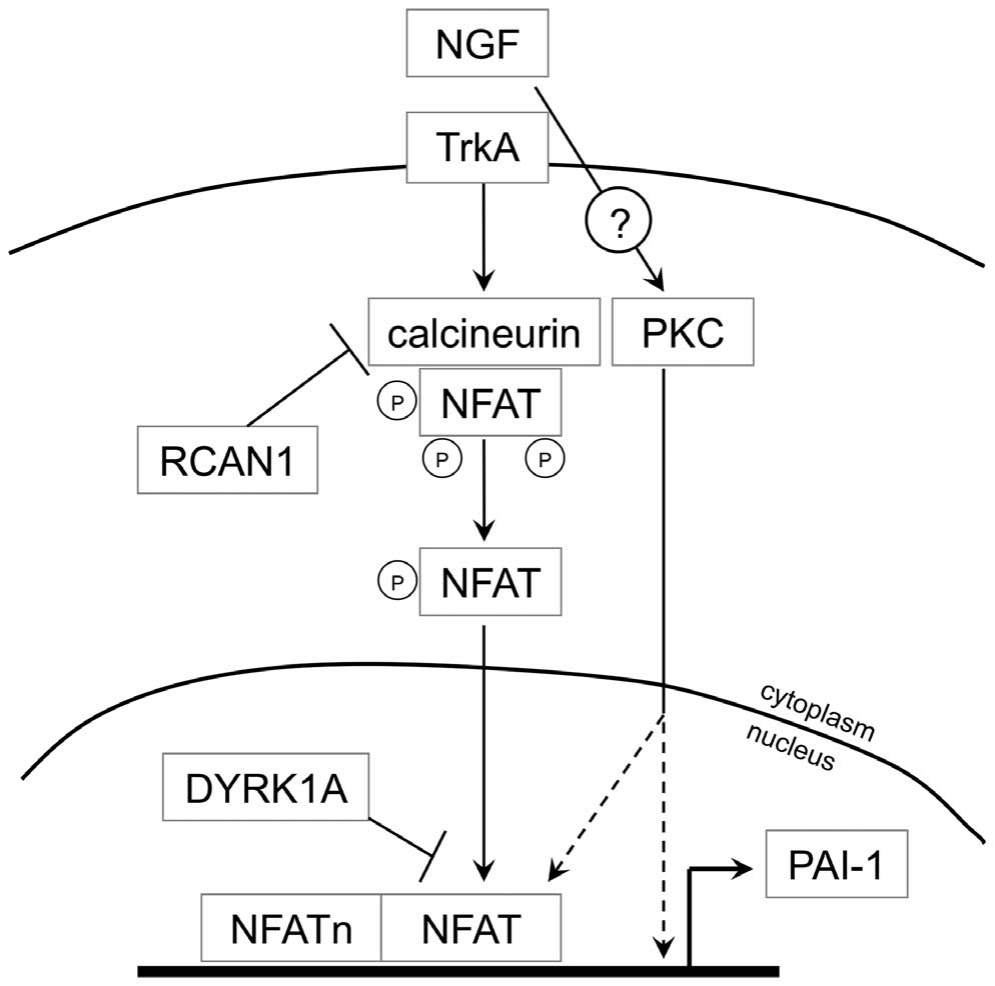
Scheme illustrating the proposed pathway by which NGF induces PAI-1 expression and its negative regulation by DYRK1A and RCAN1. NGF activates the calcineurin/NFAT pathway via the TrkA receptor and subsequently the activated NFAT induces the transcription of PAI-1. RCAN1 inhibits the dephosphorylation/activation of NFAT by the protein phosphatase calcineurin. DYRK1A reduces the NGF effect by phosphorylation/deactivation of NFAT. In parallel, NGF induces PAI-1 transcription by activating the PKC pathway. The dashed arrows suggest the possible ways of PKC implication in the induction of PAI-1 transcription.

The present results show that once NGF is bound on the TrkA receptor in PC12 cells, it upregulates PAI-1 *via* the calcineurin/NFAT pathway and that the overexpression of DYRK1A and RCAN1 partially blocks this effect of NGF (Fig. 9). Studies in Down syndrome mouse models overexpressing DYRK1A will be needed to assess whether these mechanism are also operative *in vivo* in the intact brain. Nevertheless, given that the blood PAI-1 levels in Down syndrome individuals are lower than in controls [36] we hypothesize that the attenuation of the NGF-induced PAI-1 by overexpression of DYRK1A and RCAN1 due to trisomy 21 may be relevant to several Down syndrome features. Below we want to present some considerations in this respect. Firstly, DYRK1A is downregulated during brain development as the neurons begin to migrate away from the ventricular zone and is again expressed once they reach their target positions [12]. Considering the known role of PAI-1 in cell migration [45], inhibition of the NFAT-mediated regulation of PAI-1 through DYRK1A overexpression could be involved in the abnormal neuronal migration in Down syndrome. Secondly, individuals with Down syndrome have a low risk for solid tumors, which has been suggested to be related to the negative regulation of angiogenesis by RCAN1 [64], [65]. Conversely, both PAI-1, which is used as a prognostic marker for cancer, and NGF can induce tumor angiogenesis [66], [67]. The negative effect of DYRK1A and RCAN1 on PAI-1 expression could contribute to the reduced cancer risk in Down syndrome.

### Conclusions

Here we show that NGF upregulates PAI-1 expression in young hippocampal murine cultures and that in neural PC12 cells this effect is mediated by at least two pathways, the calcineurin/NFAT and the PKC pathways. Overexpression of the Down syndrome-related proteins DYRK1A or RCAN1 attenuates the activation of NFAT by NGF and blocks the upregulation of PAI-1 expression. *In vivo* experiments need to be conducted to test the hypothesis that these newly characterized pathways may be related to the pathophysiological alterations associated with Down syndrome such as intellectual disability and altered angiogenesis in tumor development.

## Methods

### Ethics Statement

All animal procedures were approved by the local ethical committee (Comité Ético de Experimentación Animal del PRBB (CEEA-PRBB); procedure numbers MDS-08-1060P1 and JMC-07-1001P1-MDS), and met the guidelines of the local (law 32/2007) and European regulations (EU directive n° 86/609, EU decree 2001-486) and the Standards for Use of Laboratory Animals n° A5388-01 (NIH). The CRG is authorized to work with genetically modified organisms (A/ES/05/I-13 and A/ES/05/14). All efforts were done to minimize animal suffering.

### Reagents

Murine 7S NGF was obtained from Invitrogen. Rat recombinant PAI-1, calcimycin, FK506 (also known as Tacrolimus), 11R-VIVIT, calcimycin (also known as A23187) and Gö6976 were purchased from Calbiochem. ATP and doxycycline were purchased from Sigma. K252A was purchased from ENZO.

### Plasmids

A lentiviral vector system was used to generate PC12 cell lines for the inducible overexpression of DYRK1A or RCAN1. In brief, rat DYRK1A cDNA was amplified by PCR and subcloned into the lentiviral FUW-tetO backbone after removing the hMYC insert of the FUW-tetO-hMYC plasmid (Addgene 20723) by *Eco*RI digestion, resulting in the final vector FUW-tetO-DYRK1A. The plasmid pHA-CALP1L [68], which encodes the human full-length RCAN1 isoform 1.1 was kindly gifted by Dr Susana de la Luna (Center for Genomic Regulation, Barcelona). The *Eco*RI restriction site, which was located downstream of the ATG, was repositioned upstream the start codon (without disturbing the amino acid sequence) and the HA-RCAN1 fragment was obtained after digestion with *Eco*RI. The HA-RCAN1 fragment was then inserted in the FUW-tetO backbone and the final plasmid was named FUW-tetO-HA.RCAN1.

### Cell Lines and Cell Culture

The construction of PC12-NFAT-Luc cells has previously been described [37]. All PC12 subclones were grown in high glucose (4.5 g/L) Dulbecco’s modified Eagles medium (DMEM) with L-glutamine and sodium pyruvate, supplemented with 5% foetal bovine serum (PAA Laboratories), 10% horse serum (PAA) and 25 mM Hepes buffer (Sigma). Cells were grown to 60–80% confluency and cultures were split every 48–72 h without trypsinization. Lenti-X 293T cells (Clontech) were grown in high glucose (4.5 g/L) DMEM with L-glutamine and sodium pyruvate, supplemented with 10% foetal bovine serum. All cells were kept in 5% CO_2_ at 37°C in T75 filter flasks (Sarstedt).

### Virus Production and Lentiviral Transductions

4×106 Lenti-X 293T cells were plated in 10 cm collagen coated dishes. The next day the cells were transfected with 3.75 µg FUW-tetO-HA.RCAN1, FUW-tetO-DYRK1A or FUW-M2rtTA (Addgene plasmid 20342) [69] along with 1.32 µg pMD2.G (Addgene plasmid 12259) and 2.43 µg psPAX.2 (Addgene plasmid 12260) using the JetPEI transfection reagent (Polyplus). The virus containing medium was collected after 24, 48 and 72 h, filtered through a 0.20 µm syringe filter and centrifuged for 2 h at 50,000 g. Virus pellets were redissolved in PBS and used for cell transduction.

### Western Blotting

PC12 cells were plated at a density of 100,000 cells/cm2 in 12-well dishes one day before the treatments. Cells were lysed with 70 µl/well boiling SDS-buffer (20 mM Tris pH 7.4; 1% SDS). Proteins were separated by 10% SDS-PAGE, transferred to nitrocellulose membranes and blocked overnight at 4°C in Tris-buffered saline, 0.01% Tween 20 (TBS-T) containing 3% (w/v) BSA before they were incubated with the primary antibody overnight at 4°C. The following antibodies were used: rabbit polyclonal PAI-1 antibody (Abcam, #Cab7205), rabbit polyclonal phospho-PKC (pan betaII Ser660) antibody (Cell Signaling, #9371), mouse monoclonal DYRK1A antibody (Abnova, #1859), rabbit polyclonal DSCR1 antibody (Abgent, #AP6315c) or the chicken polyclonal antibody for class III beta-tubulin (Neuromics, #CH23005). The last was used as loading control since 6 h of NGF treatment did not affect its expression. The Western blots were developed using horseradish peroxidase-coupled secondary antibodies and chemiluminescence detection. Signal intensities were quantified with a LAS-3000 CCD imaging system and the AIDA Image Analyzer 5.0 program (Raytest, Straubenhardt, Germany).

### ELISA

Quantification of free, latent and complexed PAI-1 secreted into the medium was carried out by using the RPAIKT-TOT ELISA kit (Molecular Innovations), following the manufacturer’s instructions.

### Reporter Gene Assays

PC12-NFAT-Luc cells contain a stably integrated reporter gene plasmid for luciferase expression under the control of four direct repeats of the NFAT binding sequence from the IL-2 gene promoter [37]. Luciferase assays were performed for measuring of NFAT activity using the Luciferase Assay System (Promega). 200,000 cells/cm2 were plated one day before the treatments in 96-well plates. After the treatments the medium was removed and the cells were lysed in 30 µl passive lysis buffer by vigorous shaking for 1 min at room temperature. Luciferase activity was determined by mixing 20 µl lysate with 80 µl luciferase assay mix and measuring light emission in an Orion Microplate Luminometer (Berthold Detection Systems). All data were obtained from triplicate wells.

### Quantitative RT-PCR

For the quantification of the mRNA levels of PAI-1, 150,000 cells/cm2 were plated one day before the treatment in 60 mm culture dishes. Total RNA was isolated using the RNeasy Mini Kit (Qiagen) according to the manual. For cDNA synthesis 1 µg of total RNA was reverse transcribed using 1 µg of oligo(dT) primers and 2000 units of MMLV reverse transcriptase (Promega) at 40°C for 1 h. The resulting cDNAs were analysed using a LightCycler 480 system and SYBR Green master mix reagent (Roche Applied Science) using the following PCR conditions: 5 min initial denaturation at 95°C, followed by 45 cycles of 10 s at 95°C, 10 s at 50-60°C, 15 s at 72°C and 1 s at 74°C. The following oligonucleotide primers were used for the detection of the rat PAI-1 transcript: rPAI-1*for*: 5′-GAGGATGAAAGAAACAGCCAGCT-3′ and rPAI-1*rev*: 5′-CCCGCTATGAAATTAGATTCACGT-3′ [70], for the detection of the murine PAI-1 transcript: mPAI-1for: 5′-GTACTGCGGATGCCATCTTT-3′ and mPAI-1rev: 5′-GCGTGTCAGCTCGTCTACAG-3′ [71] and for the detection of the rat RCAN1-4 transcript: RCAN1-4*for*: 5′-GCCCGTTGAAAAAGCAGAAT-3′ and RCAN1-4*rev*: 5′- GACAGGGGGTTGCTGAAGTT-3′ [37]. The beta-2 microglobulin transcript was used as a housekeeping gene for normalization.

### Primary Hippocampal Cultures

Primary hippocampal cultures were prepared from embryonic day 17 mice as described by Banker and Goslin [72] with slight modifications. Briefly, pregnant C57BL/6J mice were anesthetized in a CO_2_ chamber and sacrificed by cervical dislocation, embryos were removed and hippocampi and cerebral cortices were dissected. Cells were dissociated by trituration and plated in polyethylenimine-coated 12-well plates (130,000 cells/cm2) in Neurobasal medium containing Glutamax (Invitrogen) and B27 supplement (Invitrogen).

### Immunoprecipitation and DYRK1A Kinase Assays

Cells were plated one day before the treatment in 10 cm dishes (130,000 cells/cm2). After treatment the cells were lysed in immunoprecipitation lysis buffer (50 mM Hepes pH 7.5, 150 mM NaCl, 15% (v/v) glycerol, 2 mM EDTA, 10mM NaF, 5mM NaPP, 1% (v/v) Igepal CA-630 and protease inhibitors. Lysates were precleared by incubation with Protein A Sepharose beads (Amersham) for 1 hour in a rotator at 4°C. The supernatants were rotated overnight at 4°C with 1 µg of the indicated antibodies and the next day EZview Red Protein G Affinity Gel (Sigma) was added for 2 additional hours. The beads were washed twice with immunoprecipitation buffer, once with immunoprecipitation buffer without protease inhibitors and detergent and once with kinase buffer (25 mM Hepes pH 7.5, 0.5 mM MgCl_2_, 0.5 mM dithiothreitol). The kinase assays were performed with the biotinylated peptide SAPtide (Bio-RRARKLTATPTPLGG) as described previously [73]. In brief, immunocomplexes were incubated for 30 min at 30°C in 20 µl of kinase buffer, at a final concentration of 10 µm ATP and (γ-32P)ATP (100–150 mCi/pmol) and with 100 µm of the peptide. Incorporation of 32P into SAPtide was determined with the help of the SAM2 biotin capture membrane according to the manufacturer’s instructions (Promega).

### Determination of Neurite Outgrowth

PC12 cells were plated in collagen treated 6-well plates (5,200 cells/cm2) in growth medium. After 2 h the medium was replaced by DMEM without supplements and the next day by growth medium containing NGF (50 ng/ml) in the presence/absence of doxycycline. PC12 cell differentiation was determined 2 days later when differentiation was not yet completed to facilitate detection of treatment differences. Neurite length was scored by bright field microscopy. Cells having one or more neurites of a length greater than 1.5-fold the diameter of the cell body were scored as positive. At least 200 cells were counted for each treatment. Data were obtained from three independent experiments.

### Statistical Analysis

All data derived from three independent experiments, except data shown in figure 2C. The GraphPad Prism 5.0 program (GraphPad Software, La Jolla, CA) was used for statistical analysis. Results were tested for statistical significance by ANOVA, Student’s t test or One sample t-test as appropriate.
